# Six steps in quality intervention development (6SQuID)

**DOI:** 10.1136/jech-2015-205952

**Published:** 2015-11-16

**Authors:** Daniel Wight, Erica Wimbush, Ruth Jepson, Lawrence Doi

**Affiliations:** 1 MRC/CSO Social and Public Health Sciences Unit, University of Glasgow, Glasgow, UK; 2 Evaluation Team, NHS Health Scotland, Edinburgh, UK; 3 MRC/CSO Scottish Collaboration for Public Health Research and Policy, University of Edinburgh, Edinburgh, UK

**Keywords:** PUBLIC HEALTH, PREVENTION, HEALTH PROMOTION, GENDER

## Abstract

Improving the effectiveness of public health interventions relies as much on the attention paid to their design and feasibility as to their evaluation. Yet, compared to the vast literature on how to evaluate interventions, there is little to guide researchers or practitioners on how best to develop such interventions in practical, logical, evidence based ways to maximise likely effectiveness. Existing models for the development of public health interventions tend to have a strong social-psychological, individual behaviour change orientation and some take years to implement. This paper presents a pragmatic guide to six essential Steps for Quality Intervention Development (6SQuID). The focus is on public health interventions but the model should have wider applicability. Once a problem has been identified as needing intervention, the process of designing an intervention can be broken down into six crucial steps: (1) defining and understanding the problem and its causes; (2) identifying which causal or contextual factors are modifiable: which have the greatest scope for change and who would benefit most; (3) deciding on the mechanisms of change; (4) clarifying how these will be delivered; (5) testing and adapting the intervention; and (6) collecting sufficient evidence of effectiveness to proceed to a rigorous evaluation. If each of these steps is carefully addressed, better use will be made of scarce public resources by avoiding the costly evaluation, or implementation, of unpromising interventions.

## Introduction

Improving the effectiveness of public health interventions depends as much on improving their design as their evaluation.^1^ Yet, compared to the vast literature on intervention evaluation,^2–5^ there is little to guide researchers or practitioners on developing interventions in logical, evidence-based ways to maximise effectiveness. Poor intervention design can waste public resources through expensive evaluation or, worse, the implementation of ineffective interventions unevaluated.

Existing frameworks and guidance for the development of interventions^3^ ^4^ ^6–10^ are summarised in table 1. These tend to be orientated towards social-psychological, individual behaviour change and either provide little specific detail on intervention development or require great technical skills and resources. Drawing on the strengths of these existing frameworks and our own experiences, this article outlines a pragmatic six-step guide to the essential stages of intervention development to assist public health practitioners and researchers. The focus is on public health interventions, although the model should have wider applicability.

**Table 1:** Existing frameworks and guidance for public health intervention development

Guidance/framework	Description	Possible limitations
Intervention mapping^6^	Extremely rigorous and elaborate approach to intervention development through six steps	Individual, social-psychological orientation. Highly technical, prescriptive, can require years to implement, difficult to operationalise
Conceptual framework for planning intervention-related research^7^	Specifies nine steps in developing and evaluating public health interventions	Insufficient detail in steps for operationalising
PRECEDE–PROCEED model^8^	Socioecological approach. Planning phase is PRECEDE; evaluation is PROCEED. Extensively data driven and practical application	May require great technical skill, time and money. Little detail on intervention development
Framework for design and evaluation of complex interventions to improve health.^9^	Useful guide to development of interventions within the context of healthcare services	Focus on healthcare not public health. Little detail on intervention development
MRC guidance for the development and evaluation of Complex interventions^4^	Identifies three broad stages of intervention development: developing theory, modelling process and outcomes and assessing feasibility	Does not break down three stages any further. Primarily devoted to evaluation
Design for behaviour change framework^10^	For community development workers in low income countries. Focuses on determinants, facilitators and barriers to intended behaviour to plan behaviour change projects strategically	Sequence of steps advocated in part illogical and some of the terminology confusing

A public health intervention is defined as planned actions to prevent or reduce a particular health problem, or the determinants of the problem, in a defined population. Most require some level of social interaction. They are rarely simple, singular actions that can be easily replicated but more often complicated (multicomponent) or complex programmes (with feedback loops and emergent outcomes)^11^ that are designed to affect change at several levels of the socioecological model^12^ (table 2). By and large, ‘upstream’ interventions ‘require less individual effort (from recipients) and have the greatest population impact’,^13^ ^14^ whereas interventions requiring voluntary uptake are more likely to exacerbate health inequalities.^15^

**Table 2:** Examples of interventions, mechanisms and outcomes at different levels

Level	Interventions	Change mechanisms	Outcomes
Individual	Information provision; advertising	Resonance; perceived relevance; reading and reflection	Improving knowledge, motivation/intentions
Interpersonal	Counselling; peer education	Modelling; influence of reference group; mentorship	Improving motivation/intentions; developing skills/self-efficacy
Community	Walking group; food co-operation; neighbourhood watch	Solidarity; diffusion of innovation; changing community norms	Improving motivation/intentions, physical activity, diet, sense of security
Organisational	Institutional policies; quality standards; partnership working	Authorisation; inspection; enforcement; increasing staff awareness	Reducing discrimination; improving services
Environmental	Clean air legislation; piped water; housing regulations; safe cycling and walking infrastructure	Legislation; enforcement; redesign of services; ‘choice architecture’	Environmental improvements; healthier housing; more physical activity
Macro policy	Poverty reduction; redistribution of resources; education; controlling corporations	Legislation and enforcement; economic security and choices	Healthy lifestyles more affordable and given higher priority

Interventions are best developed through collaborations between interdisciplinary teams of practitioners, researchers, the effected population and policymakers. Such coproduction maximises the likelihood of intervention effectiveness by improving: the fit with the target group's perceived needs and thus acceptability; practicality; evaluability, including the theorising of causal pathways; and uptake by practitioners and policymakers.

This paper sets out six crucial steps in the development of public health interventions (box 1). For an illustrative case study we use the early prevention of gender-based violence (GBV) in Uganda through a parenting intervention that addresses familial predictors of GBV. However, we also draw on other relevant examples to illustrate key points.^16^ ^17^

Box 1 **Main steps in public health intervention development**

1. Define and understand the problem and its causes.
2. Clarify which causal or contextual factors are malleable and have greatest scope for change.
3. Identify how to bring about change: the change mechanism.
4. Identify how to deliver the change mechanism.
5. Test and refine on small scale.
6. Collect sufficient evidence of effectiveness to justify rigorous evaluation/implementation.

### 1. DEFINE AND UNDERSTAND THE PROBLEM AND ITS CAUSES

Our starting point is that a public health problem has already been identified as requiring intervention. Often this results from a needs assessment, for which there are several practical guides,^8^ ^18^ or from a political process such as a manifesto commitment.

Clarifying the problem with stakeholders, using the existing research evidence, is the first step in intervention development. Some health problems are relatively easily defined and measured, such as the prevalence of a readily diagnosed disease, but others have several dimensions and may be perceived differently by different groups. For instance, ‘unhealthy housing’ could be attributed to poor construction, antisocial behaviour, overcrowding or lack of amenities. Definitions therefore need to be sufficiently clear and detailed to avoid ambiguity or confusion. Is ‘the problem’ a risk factor for a disease/condition (eg, smoking) or the disease/condition itself (eg, lung cancer)? If the former, it is important to be aware of the factor's importance relative to other risk factors. If this is modest even a successful intervention to change it might be insufficient to change the ultimate outcome.

Once defined one should try to establish how the problem is socially and spatially distributed, including who is currently most/least likely to benefit from an intervention. It is also important to consider what interventions or policies currently exist and why they are not deemed adequate.

**Figure 1:**
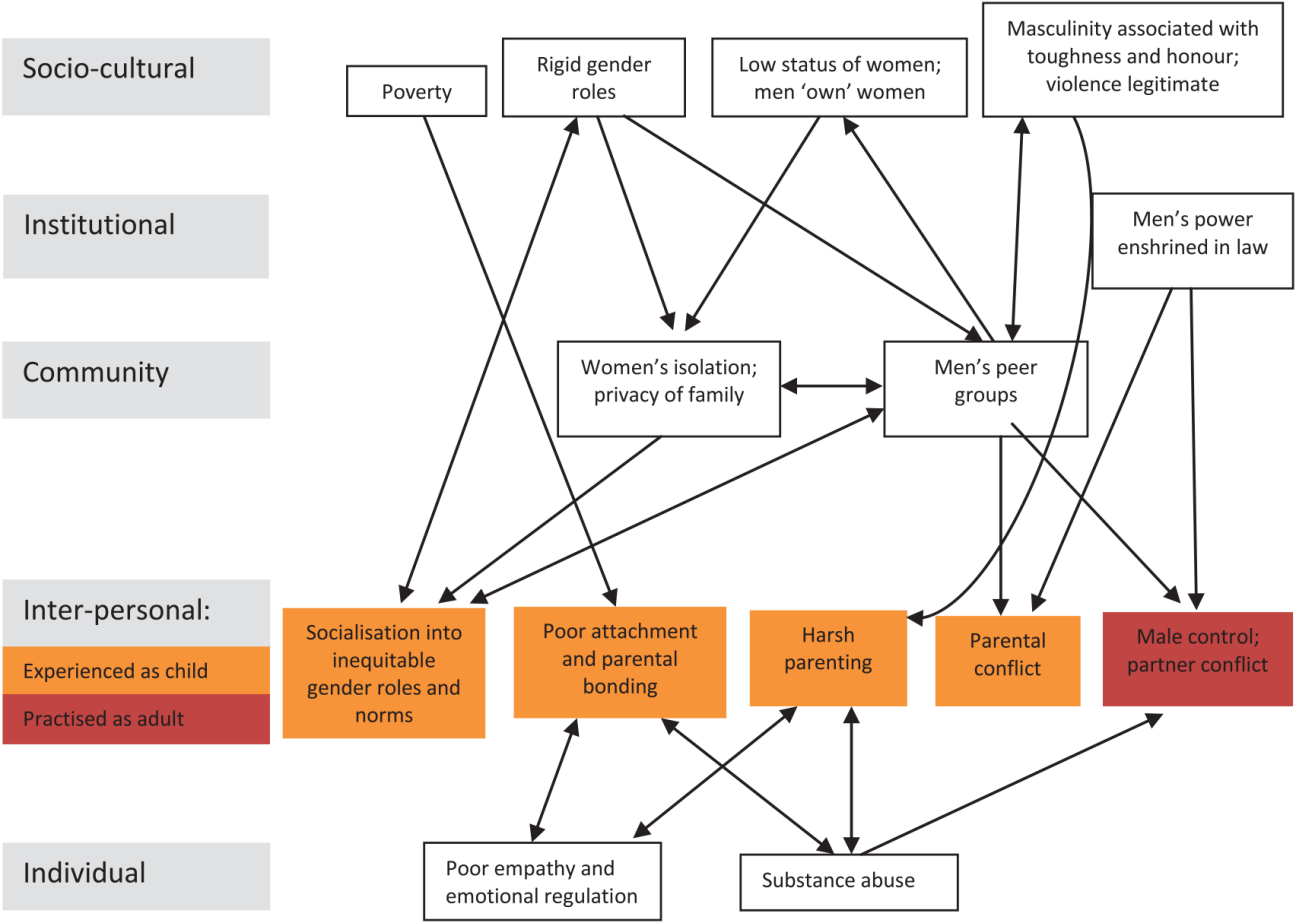
Causal pathways perpetuating gender-based violence.^21–26^

Having defined the problem, one needs to understand, as far as possible, what are the immediate (proximal) and underlying (distal) influences that give rise to it. These are often suggested by the distribution of the problem, its history and relationship to the life course. It is only by understanding what shapes and perpetuates the problem (the causal pathways) that one can identify possible ways to intervene. Case study step 1 applies Funnell and Rogers’ useful questions for problem analysis to GBV (ref. 19, p. 160). The main influences on the problem can also be classified according to the socioecological model.^12^ ^20^ It can often be helpful to present the various causal pathways affecting the problem diagrammatically: figure 1 attempts to do this for GBV, distinguishing different levels of the socioecological model.

**Table:** 

Case study step 1: Understanding the problem of GBV and its causes^21–26^	
Questions	Answers
Nature and extent of main problem	
What is the nature and extent of problem?	1 in 3 women likely to experience GBV
For whom and at what levels does problem exist?	Predominantly affects girls and women. Problem perpetuated at different socioecological levels
What is the history?	Embedded in long-established patriarchal institutions and norms but these are weakening with improved education, employment and rights for women
Causes and contributing factors	
What are its causes?	*Individual*: poor empathy and emotional regulation, substance abuse. *Interpersonal*: as child—poor attachment and parental bonding, socialisation into gender roles and norms, harsh parenting, witnessing parental conflict; as adult—marital conflict, male control of wealth and decision-making. *Community:* women's isolation, men's peer group acceptance of violence. *Sociocultural*: rigid gender roles and norms, masculinity linked with toughness and honour, men's power enshrined in law
Which causes are most important? (see case study step 2)	Poor attachment and parental bonding; harsh parenting; witnessing parental conflict
What has been effective in addressing problem?	Early years parenting programmes; relationship counselling; mediation; empowering women educationally and economically; changed norms; legislation
Consequences	
What are the consequences for those directly affected?	*Immediate*: fear, injuries, sexually transmitted infections, unintended pregnancies and death. *Long term*: low self-esteem, depression and drug abuse
What are the consequences for those indirectly affected?	Those witnessing more likely to become victims or perpetrators; family break-up; burden on health services; women unable to fulfil potential

### 2. CLARIFY WHICH CAUSAL OR CONTEXTUAL FACTORS ARE MALLEABLE AND HAVE GREATEST SCOPE FOR CHANGE

The next step is to identify which of the immediate or underlying factors that shape a problem have the greatest scope to be changed. These might be at any point along the causal chain. For example, is it more promising to act on the factors that encourage children to start smoking (primary prevention), or to target existing smokers through smoking cessation interventions (secondary prevention)? In general, ‘upstream’ structural factors take longer and are more challenging, to modify than ‘downstream’ proximal factors, but if achieved structural changes have greatest population impact, as noted above.^13^

With complex problems the causal pathways can be very diverse and interwoven. If they have been described diagrammatically in step 1, it will be easier to identify where one might intervene and, critically, whether it is necessary to intervene at more than one point, or on more than one level, to interrupt the most important causal pathways. One must also assess which changes would have most effect. Most interventions take place within systems (eg, healthcare, education, criminal justice) and exert their influence by changing relationships, displacing existing activities and redistributing and transforming resources.^27^ It is necessary, therefore, to consider which system an intervention would operate in, how the system is likely to interact with the intervention and whether that system needs to be/can be modified as well. For example, a school-based intervention to improve pupils’ social and emotional well-being is likely to be affected by existing school structures, relationships and timetables and might require their modification. Interventions that address complex problems through multilevel actions are more likely to maximise synergy and long-term success.^13^ The potential different levels of intervention are shown in table 2.

In the case study of GBV, it was decided to focus on early prevention in families since: this has the potential for widespread, long-term change; it may improve other outcomes; and more proximal factors were already being addressed.

**Table:** 

Case study step 2: Modifiable familial factors shaping GBV	
Factor	Evidence modifiable
1. Poor attachment and parental bonding	Effective early years parenting programmes; historical change
2. Harsh parenting	ditto
3. Socialisation into inequitable gender roles and norms	Historical change
4. Parental conflict	Effective relationship counselling and mediation; historical change

### 3. IDENTIFY HOW TO BRING ABOUT CHANGE: THE CHANGE MECHANISM

Having identified the most promising modifiable causal factors to address, the next step is to think through how to achieve that change. All interventions have an implicit or explicit programme theory^19^ about how they are intended to bring about the desired outcomes. Central to this is the ‘change mechanism’ or ‘active ingredient’,^2^ the critical process that triggers change for individuals, groups or communities (see table 2).

It is usually helpful to depict the programme theory diagrammatically (see http://www.theoryofchange.org/). Many interventions are not intended to achieve the final goal directly, but have short-term and intermediate outcomes that are expected to lead to the long-term outcomes. Ideally a range of stakeholders are involved in formulating the programme theory A common pitfall is that it is wildly optimistic, with little empirical evidence to support each link in the causal chain. For instance, a short-term change in health-related knowledge may be necessary, but it is rarely sufficient to achieve behaviour change let alone prevent the disease in question.

The best developed programme theories are based on formalised theories of behaviour change (eg, Social Cognition Theory^28^ or the Theory of Reasoned Action^29^). This is not essential, but can be very helpful if the theory has strong predictive and explanatory power. However, not many do,^30^ ^31^ perhaps because they often only address one causal strand (cognitions or motivation) and not socioenvironmental determinants. Furthermore, few interventions said to be based on such formalised theories clarify how the theory has been operationalised. What is crucial in intervention development is that the change mechanisms in the programme theory are clearly articulated.

The interpersonal change mechanisms for the GBV case study are shown below (there is not space to show those at the community level). Critical to their effectiveness is who delivers the intervention and their relationship with the target group.

**Table:** 

Case study step 3: Change mechanisms for early prevention of GBV (interpersonal level only)		
Modifiable factors	Change mechanisms	Is this sufficient to reduce the problem?
1. Poor attachment and parental bonding	Explaining infant development and parent–child interactions	Probably
2. Harsh parenting	Praising and reinforcing parents’ techniques of positive parenting	Not if poor attachment
3. Socialisation into inequitable gender roles and norms	Raising awareness of gender issues and discussing between couples	Not if poor attachment and harsh parenting impair emotional control
4. Parental conflict	Understanding impact of parental conflict on child development and well-being Developing parents’ skills to sustain and improve relationships with partner Strengthening motivation to reduce parental conflict Mediating mothers’ and fathers’ views on parenting	Probably

### 4. IDENTIFY HOW TO DELIVER THE CHANGE MECHANISMS

Having identified the change mechanisms, step 4 requires working out how best to deliver them. As with other steps, it is helpful to involve stakeholders with the relevant practical expertise to develop the implementation plan. Sometimes change mechanisms can only be brought about through a very limited range of activities, for instance legal change is achieved through legislation. However, other change mechanisms might have several delivery options; for instance modelling new behaviours could be performed by teachers, peers or actors in TV/radio soap operas. The choice is likely to be target group-specific and context-specific.

The implementation plan requires clarifying the conditions and resources necessary for successful implementation and the related risks and assumptions. For example, if an intervention is to be delivered by health visitors, are they available everywhere and will their senior managers allow time for training and delivery? In low income countries resource constraints can seriously restrict options for delivery, for instance the existence of suitably skilled facilitators, or an ethos of voluntarism. In step 4 one should also anticipate possible unintended effects of the intervention and minimise any that might be harmful. These have been categorised by Lorenc and Oliver^32^ as fivefold: direct, psychological, equity, group/social and opportunity. ‘Equity harms’ are currently of particular policy concern, the greatest beneficiaries of many behaviour change interventions being the higher educated or more affluent, thereby exacerbating inequalities in health outcomes.^15^

The delivery of the change mechanisms for our case study of GBV is set out below.

**Table:** 

Case study step 4: Delivering change mechanisms for early prevention of GBV
1. Identify suitable villages or urban wards
2. Explain programme and offer it to community leadership: awareness raising critical
3. Recruit existing groups or ‘opinion leaders’ and form parent groups
▸ initially single sex
▸ groups select facilitator
4. Deliver 2 weeks training to facilitators
5. Facilitators lead 10 weekly single sex sessions of about 2 h
6. Following five sessions facilitators recruit novice facilitator to mentor
7. After 10 sessions groups split in half and pair up with group of opposite sex
8. Facilitators lead 11 mixed sex sessions
▸ groups explore different understandings of parenting and GBV
9. Groups present recommendations for village/ward level to village/ward leaders
10. Trained facilitators start new groups and recruit others to be trained as facilitators

### 5. TEST AND REFINE ON SMALL SCALE

Once the initial intervention design has been resolved, in most cases its feasibility needs to be tested and adaptations made. This varies considerably according to the type of intervention. For instance, national legislation or large-scale health protection measures, such as water fluoridation, are difficult to pilot before full implementation. Phased region by region implementation might allow incremental adjustments, but the scope for adaptation is primarily around implementation rather than the mechanism of change. With individual or community level interventions a long process of repeated testing and adaptation is often required, often called ‘formative evaluation’, especially if the intervention is novel or highly innovative.

Testing the intervention can clarify fundamental issues such as: acceptability to the target group, practitioners and delivery organisations; optimum content (eg, how participatory), structure and duration; who should deliver it and where; what training is required; and how to maximise population reach.

Frequently this is the most hurried stage of intervention development, due to lack of resources and time, but this often compromises subsequent effectiveness. Ideally incremental adaptations would each be tested separately, but in practice adaptations can be made simultaneously if sufficiently rich data are collected to enable judgements about which are helpful and which not. Practical constraints eventually force the decision that the intervention is ‘good enough’ to go to the next step. The testing and adapting of the GBV programme is set out below.

**Table:** 

Case study step 5: Testing and adapting programme for early prevention of GBV
▸ Negotiate access to village and recruit one mothers and one fathers group
▸ Recruit and train two facilitators from each group
▸ Pilot draft manual with groups with observational research
▸ Revise problematic sessions as necessary and test again (if necessary several times)
▸ Conduct group discussions with each group and in-depth interviews with facilitators to explore views on intervention
▸ Finalise first draft of manual
▸ Repeat process in second village with two mothers and two fathers groups to produce second draft of manual

## 6. Collect sufficient evidence of effectiveness to justify rigorous evaluation/implementation

Before committing resources to a large scale rigorous evaluation (typically a ‘phase III’ RCT), the final step is to establish sufficient evidence of effectiveness to warrant such investment. Beyond the research world, especially in third sector organisations, inadequate resources often mean practitioners move to wide scale implementation without such rigorous evaluation. This makes step 6 all the more critical.

What is being sought at this stage is some evidence that the intervention is working as intended, it is achieving at least some short-term outcomes, and it is not having any serious unintended effects, for instance exacerbating social inequalities. It is unlikely that the evaluation design will seek to prove causality so theory-based evaluation approaches are likely to be most appropriate. There are numerous guides on evaluation that adequately cover step 6,^4^ ^33^ but it is worth re-stating that often the most practical way to collect evidence of effectiveness with limited resources is through a before and after survey, or by using routinely collected data. If possible, a control group greatly increases the strength of evidence. If a phase III RCT is planned, an ‘exploratory trial’ can provide valuable information about the acceptability of evaluation designs, appropriate measures and likely effect sizes to inform subsequent trials. Plans for this final step with our case study are set out below.

**Table:** 

Case study step 6: Collecting evidence of effectiveness of programme for early prevention of GBV.
▸ Baseline survey of parent and 10–14-year-old child dyads in two communities to measure: attitudes to GBV, parent–child relationships, parent–parent relationships, etc
▸ Implement intervention with 10 different groups
▸ Observe 10% of sessions to assess fidelity
▸ Group discussions and semistructured interviews with participants to assess their characteristics, recruitment processes, group functioning and requirements for facilitators
▸ Follow-up survey, ideally with the same respondents

## Conclusion

In order to improve the effectiveness of public health interventions, a systematic approach to intervention development is required, as well as rigorous evaluation. However, little practical guidance exists for public health practitioners and researchers that explains the essential stages of intervention development. We argue that this process can be broken down into six key steps: defining and understanding the problem; identifying modifiable determinants; deciding on the mechanisms of change; clarifying how these will be delivered; testing and adapting the intervention; and collecting initial evidence of effectiveness. This model imposes somewhat arbitrary cut-offs in the process of intervention development and suggests a linear progression. In practice developers often return to an earlier step in the sequence before reaching step 6, and subsequently ‘definitive trials’ can lead to further revisions of the intervention. However, we hope that if each of these six steps is carefully addressed in the design of interventions better use will be made of scarce public resources by avoiding the costly evaluation, or implementation, of unpromising interventions.

**What is already known on this subject?**

- There is little practical guidance for researchers or practitioners on how best to develop public health interventions. Existing models are generally orientated towards individual behaviour change and some are highly technical and take years to implement.

**What this study adds?**

- This paper provides a pragmatic six-step guide to develop interventions in a logical, evidence-based way to maximise likely effectiveness. If each step is carefully addressed, better use will be made of scarce public resources by avoiding the costly evaluation, or implementation, of unpromising interventions.
